# Systolic Longitudinal Function of the Left Ventricle Assessed by Speckle Tracking in Heart Failure Patients with Preserved Ejection Fraction 

**Published:** 2015-10-27

**Authors:** Mehrnoush Toufan, Saeed Mohammadzadeh Gharebaghi, Leili Pourafkari, Elham Delir Abdolahinia

**Affiliations:** 1 *Cardiovascular Research Center, Tabriz University of Medical Sciences, Tabriz, Iran.*; 2 *Bone Research Center, Tabriz University of Medical Sciences, Tabriz, Iran. *

**Keywords:** *Echocardiography*, *Heart failure*, *Heart ventricles*

## Abstract

**Background: **Echocardiographic evaluations of the longitudinal axis of the left ventricular (LV) function have been used in the diagnosis and assessment of heart failure with normal ejection fraction (HFNEF). The evaluation of the global and segmental peak systolic longitudinal strains (PSLSs) by two-dimensional speckle tracking echocardiography (STE) may correlate with conventional echocardiography findings. We aimed to use STE to evaluate the longitudinal function of the LV in patients with HFNEF.

**Methods: **In this study, 126 patients with HFNEF and diastolic dysfunction and 60 normal subjects on conventional echocardiography underwent STE evaluations, including LV end-diastolic and end-systolic dimensions; interventricular septal thickness; posterior wall thickness; LV volume; LV ejection fraction; left atrial volume index; early diastolic peak flow velocity (𝐸); late diastolic peak flow velocity (𝐴); 𝐸/𝐴 ratio; deceleration time of 𝐸; early diastolic myocardial velocity (e′); late diastolic myocardial velocity (A′); systolic myocardial velocity (S); and global, basal, mid, and apical PSLSs. The correlations between these methods were assessed.

**Results:** The mean age was 57.50 ± 10.07 years in the HFNEF patients and 54.90 ± 7.17 years in the control group. The HFNEF group comprised 69.8% males and 30.2% females, and the normal group consisted of 70% males and 30% females. The global, basal, mid, and apical PSLSs were significantly lower in the HFNEF group (p value < 0.001 for all). There was a significant positive correlation between the global PSLS and the septal e' (p value < 0.001). There was a negative correlation between the global PSLS and the E/e' ratio (p value = 0.001). There was a significant negative correlation between the E/e' ratio and the mid PSLS (p value = 0.002) and the basal PSLS (p value = 0.001). There was a weak positive correlation between the septal e' and the mid PSLS (p value = 0.001) and the basal PSLS (p value < 0.001). There were also weak negative correlations between the isovolumic relaxation time and the global PSLS (p value = 0.022) and the mid PSLS (p value = 0.018) and also between the New York Heart Association functional class and the mid PSLS (p value = 0.041) and the basal PSLS (p value = 0.009).

**Conclusion: **Our HFNEF patients on conventional echocardiography had different STE findings compared to our normal subjects, which is indicative of diastolic dysfunction. The longitudinal systolic function of the LV, which was measured by STE, was reduced in all the segments, denoting some degree of subclinical systolic dysfunction in these patients.

## Introduction

Patients with signs and symptoms of heart failure (HF) with a normal left ventricular ejection fraction (LVEF) are considered as diastolic heart failure with normal ejection fraction (HFNEF). This entity represents almost half of the HF population, especially among females and older subjects,^1^^-^^4^ and is reported to have similar prognosis and mortality compared to heart failure patients with reduced ejection fraction (HFREF).^5^ Dyspnea is the common clinical manifestation in outpatient HFNEF cases.^6^^-^^8^ Patients with HFNEF, compared to patients with HFREF, are more frequently female, old, obese, diabetic, and hypertensive.^8^ These risk factors and the presence of a normal LVEF may cause misdiagnosis and lead to inadequate treatment.^9^

HFNEF patients have few differences with HFREF patients in terms of clinical signs and symptoms and radiographic findings, and echocardiography is the only accurate method for differentiating these patients.^9^ The systolic function of the left ventricle (LV) is usually measured using conventional echocardiography along with tissue Doppler. Two-dimensional (2D) speckle tracking echocardiography (STE) could be beneficial in the evaluation of the LV diastolic and systolic functions. Tissue Doppler is affected by different factors such as angle dependency, preload, and afterload and, as such, may have some limitations in the accurate assessment of the LV function.^10^^-^^15^ STE has overcome some of these shortcomings, and previous studies have assessed its accuracy and clinical utility. STE uses 2D standard images and affords an offline analysis of the previously recorded data. Considering these factors, STE has emerged as an acceptable method in echocardiographic evaluations.^16^^-^^19^

The echocardiographic evaluation of the longitudinal function of the LV has been helpful in the understanding of HFNEF.^10^^, ^^14^^, ^^20^ It has been reported that HFNEF patients have reduced LV systolic longitudinal function, which is indicative of the subclinical impairment of the systolic function.^21^^-^^24^ The use of STE seems to be useful in understanding HFNEF and making early diagnosis. In this study, we aimed to assess the utility of STE in the evaluation of the systolic longitudinal function of the LV in HFNEF patients.

## Methods

In this cross-sectional study, 126 patients with confirmed HFNEF and 60 normal subjects were enrolled. The two groups were matched for age and sex. Patients were considered to have HFNEF if they had HF symptoms and LV diastolic dysfunction with a preserved LVEF (LVEF ≥ 50%) diagnosed by conventional echocardiography according to the guidelines of the American Society of Echocardiography (ASE) and the European Association of Echocardiography (EAE).^25^^-^^27^ Patients with non-sinus rhythm (including atrial fibrillation); extensive regional wall motion abnormality at rest; LVEF < 50%; unstable vital signs; moderate to severe valvular heart disease; primary or secondary increase in pulmonary pressure not related to HFNEF or diastolic dysfunction; severe systemic disease, including pulmonary, renal, and hepatic disease; pericardial disease; cardiomyopathy; or congenital heart disease were excluded. Normal subjects without symptoms or history of cardiovascular disease, high blood pressure, and diabetes mellitus as well as normal electrocardiography and echocardiography were included as the control group. The study protocol was approved by the Ethics Committee of Tabriz University of Medical Sciences, and all the subjects gave written informed consent.

All the subjects underwent conventional echocardiography and then STE (2D strain) using the Automated Function Imaging (AFI) software on a commercially available machine (GE Vivid 7 Dimension, Norway). All the images were obtained from the standard parasternal and apical positions using 2D, M-mode, and Doppler echocardiographic techniques. The LV end-diastolic and end-systolic dimensions as well as the interventricular septal thickness (IVST) and the posterior wall thicknesses (PWT) were obtained via M-mode echocardiography.^25^ The LV volumes and the LVEF were measured using the modified biplane Simpson method as recommended by the ASE and the EAE.^26^^, ^^27^ The left atrial volume index (LAVI) values were calculated using the area length method.^28^

The pulse Doppler sample volume was placed at the tips of the mitral valve leaflets in the apical four-chamber view to record the LV inflow velocity. From the LV inflow velocity, the early diastolic peak flow velocity (E), late diastolic peak flow velocity (A), E/A ratio, and deceleration time of the E wave velocity were measured. Additionally, the early diastolic myocardial velocity (e′), late diastolic myocardial velocity (A′), and systolic myocardial velocity (S) were obtained using STE as the average of the septal lateral corner of the mitral valve. The isovolumic relaxation times were also calculated using STE.^27^

**Figure 1:**
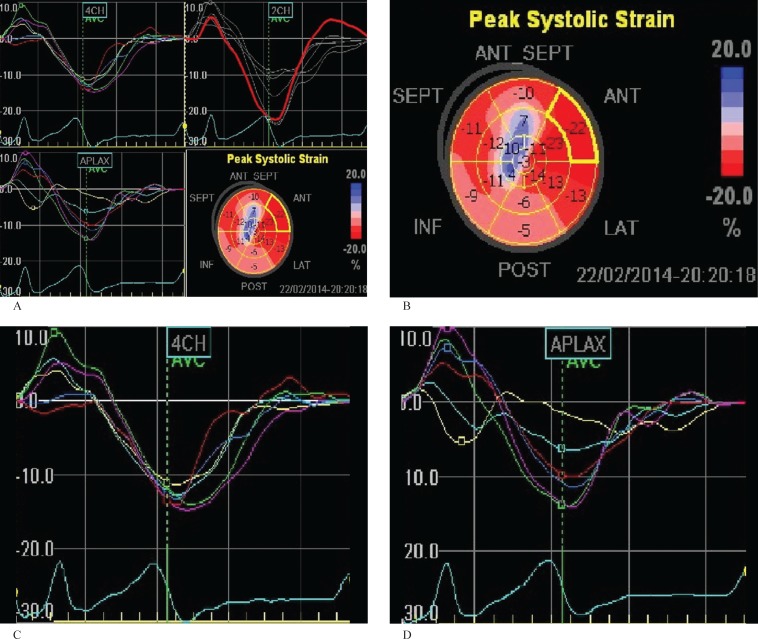
Bull’s eye and longitudinal strain curves in the four-chamber, two-chamber, and apical long-axis views in two*-*dimensional speckle tracking echocardiography*.*

The STE images of the LV were acquired in the apical four-chamber, two-chamber, and apical three-chamber views with the same ultrasound machine (Figure 1). Three consecutive cardiac cycle loops were recorded at end expiration. The frame rate was kept between 70 Hz and 100 Hz. The longitudinal strains were quantified in a 16-segment model using a novel speckle tracking system. All the measurements were stored as digital storing system for offline analysis. The global peak systolic longitudinal strain (PSLS) was defined as an average value of the 16 PSLSs of an LV. Additionally, the basal, mid, and apical PSLSs were defined as an average value of the PSLS of each of the corresponding 6 segments (4 segments for the apex). All the examinations were performed by an experienced echocardiologist.

A) Bull’s eye and longitudinal strain curves in the four-chamber (4CH), two-chamber (2CH), and apical long-axis (APLAX) views in two*-*dimensional speckle tracking echocardiography

Strain values are decreased in the mid anteroseptal portion, as is seen in both Bull’s eye and its related curve.

B) Bull’s-eye view, displaying the strain values of all the individual segments and a value for the left ventricular longitudinal global strain

C) Longitudinal strain curves obtained by speckle tracking echocardiography in the four-chamber view

D) Impaired longitudinal strain curve obtained in the apical long-axis view

4CH, Four-chamber; 2CH, Two-chamber; APLAX, Apical long-axis; ANT_SEPT, Anteroseptal; ANT, Anterior; LAT, Lateral; INF, Inferior;

SEPT, Septal

The data were analyzed using Statistical Package for Social Sciences, version 17.0 (SPSS, Chicago, Illinois). The baseline data are reported as mean ± standard deviation (continuous data) or percentages (categorical data), depending on the data level. In order to analyze the differences between the groups in the quantitative variables, the Student *t*-test was used in those with normal distribution and the Mann–Whitney *U* test if the distribution was not normal. The association between the qualitative variables was studied using the chi-square test or the Fisher exact test. The correlation between the STE and conventional variables was evaluated using the Pearson correlation. A p value ≤ 0.05 was considered significant. 

## Results

In this study, 126 patients with HFNEF and 60 normal subjects were evaluated. The demographic findings of the groups are demonstrated in Table 1. The HFNEF group had significantly higher body mass index and systolic and diastolic blood pressures. 

The conventional echocardiography findings are depicted in Table 2. The HFNEF group had significantly higher IVST, PWT, LAVI, isovolumic relaxation time (IVRT), E/e' ratio, and deceleration time and lower peak E, E/A ratio, and septal e' than the normal subjects. Diastolic dysfunction in the HFNEF group was grade I in 120 patients and grade II in 6 patients. 

According to the STE findings, the HFNEF group also had significantly lower global, apical, mid, and basal PSLSs than the normal subjects (Table 3). 

**Table 1:** Patients characteristics*

	HFNEF Group	Normal group	P Value
Age (y)	57.50±10.07	54.90±7.17	0.074
Gender			0.925
Male	38 (30.2)	18 (30.0)	
Female	88 (69.8)	42 (70.0)	
Diabetes Mellitus	2 (15.9)	0	< 0.001
Hypertension	116 (92.1)	0	< 0.001
Hyperlipidemia	30 (23.8)	0	< 0.001
Ischemic Heart Disease	10 (7.9)	0	< 0.001
NYHA Class			
I	88 (69.8)	0	< 0.001
II	38 (30.2)	0	< 0.001
Normal	0	60 (100)	< 0.001
BMI (kg/m^2^)	26.53±3.72	25.39±2.63	0.033
Heart Rate (beat/minute)	81.61±15.31	84.70±13.26	0.186
Systolic Blood Pressure (mm Hg)	173.03±164.33	117.90±9.14	0.014
Diastolic Blood Pressure (mm Hg)	87.96±10.69	67.25±5.66	< 0.001

HFNEF, Heart failure with normal ejection fraction; NYHA, New York Heart Association; BMI, Body mass index

* Data are presented as mean±SD or n (%).

**Table 2:** Comparison of conventional echocardiography findings between the groups*

	HFNEF Group	Normal Group	P Value
Left Ventricular Ejection Fraction (%)	62.14±8.02	62.30±6.89	0.890
Interventricular Septal Thickness (mm)	11.79±2.12	8.70±1.01	< 0.001
Posterior Wall Thickness (mm)	10.69±1.76	8.50±1.12	< 0.001
Left Ventricular End-Diastolic Volume (ml)	94.41±20.46	91.25±22.40	0.342
Left Ventricular End-Systolic Volume (ml)	37.19±12.17	36.55±14.03	0.755
Left Ventricular End-Diastolic Diameter (mm)	45.49±4.38	44.50±4.74	0.163
Left Ventricular End-Systolic Diameter (mm)	30.17±4.61	29.50±3.88	0.324
Left Atrial Volume Index (ml/m^2^)	44.00±10.37	24.85±3.52	< 0.001
Isovolumic Relaxation Time (msec)	94.16±11.04	81.05±7.52	< 0.001
Peak E (m/sec)	62.26±14.66	84.95±12.03	< 0.001
E/A Ratio	0.76±0.15	1.31±0.09	< 0.001
Septal e' (m/sec)	6.17±1.82	12.90±1.45	< 0.001
E/e' Ratio	10.27±2.69	6.57±0.82	< 0.001
Deceleration Time (msec)	225.69±61.81	190.00±13.09	< 0.001

HFNEF: Heart failure with normal ejection fraction; Peak E, Peak of early diastolic mitral flow velocity; A, Late diastolic peak flow velocity; Septal e', Early diastolic septal annular velocity

* Data are presented as mean±SD.

**Table 3:** Comparison of two-dimensional speckle tracking echocardiography findings between the groups*

	HFNEF Group	Normal Group	P Value
Global PSLS (%)	-17.31±3.54	-20.63±1.79	< 0.001
Apical PSLS (%)	-17.66±7.09	-22.00±3.56	< 0.001
Mid PSLS (%)	-16.04±4.88	-20.13±1.64	< 0.001
Basal PSLS (%)	-14.75±5.71	-19.20±0.52	< 0.001

HFNEF, Heart failure with normal ejection fraction; PSLS, Peak systolic longitudinal strain

* Data are presented as mean±SD.

The correlation between the STE and conventional echocardiography findings was evaluated in the HFNEF group. There was a significant positive correlation between the global PSLS and the septal e' (r = 0.36; p value < 0.001) and a negative correlation between the global PSLS and the E/e' ratio (r = -0.30; p value = 0.001). There were also significant negative correlations between the mid PSLS and the E/e' ratio (r = -0.26; p value = 0.002) and between the basal PSLS and the E/e' ratio (r = -0.28; p value = 0.001). There were weak positive correlations between the septal e' and the mid PSLS (r = 0.12; p value = 0.001) and the basal PSLS (r = 0.29; p value < 0.001). 

Our results also revealed negative correlations between the IVRT and the global PSLS (r = -0.19; p value = 0.029) and the mid PSLS (r = -0.21; p value = 0.017). The New York Heart Association (NYHA) functional class also had a weak negative correlation with the mid PSLS (r = -0.18; p value = 0.042) and the basal PSLS (r = -0.23; p value = 0.009). 

The correlation analysis was performed in the control group, but no significant correlation was observed.

## Discussion

Diastolic dysfunction with no signs and symptoms is usually present in 40%-60% of patients with coronary artery disease, hypertension, valvular heart disease, diabetes mellitus, hypertrophic cardiomyopathy, or cardiac amyloidosis and can progress to symptomatic HF.^6^^, ^^7^ The longitudinal function of the LV is a sensitive index in detecting early changes in the LV function with pathologic processes or aging. Recent studies have recommended the global PSLS as an early marker of the subclinical dysfunction of the LV.^29^^-^^31^

Diastolic dysfunction is a known predictor of the clinical outcome in many situations such as HFNEF and HFREF.^32^^, ^^33^ In the present study, we evaluated systolic function by STE in HFNEF patients with diastolic dysfunction. Diastolic dysfunction was grade I in 120 patients and grade II in 6 patients. We observed that the E/e' ratio and the deceleration time, as the indicators of diastolic dysfunction, were significantly higher in the HFNEF patients with diastolic dysfunction than the normal subjects without diastolic dysfunction. Previous studies have demonstrated that some degree of systolic dysfunction is present in HFNEF patients with diastolic dysfunction.^34^^-^^39^ Similar results were observed in the present study. 

Our results demonstrated that most of the HFNEF patients had a high LAVI with grade I diastolic dysfunction: this is considered as grade Ia diastolic dysfunction. In most patients with diastolic dysfunction grade I, the diastolic filling pressure is not increased and the E/e' ratio is 8 or higher. However, in a subgroup of patients, the E/e' ratio is > 15, with an E/A ratio < 1. This pattern has been designated grade Ia diastolic dysfunction to emphasize the fact that the filling pressure is increased, while there is a typical grade 1 mitral inflow velocity pattern. Also in this pattern, the LAVI is elevated.^40^

Morris et al.^36^ observed that their HFNEF patients with impaired systolic and diastolic functions of the LV myocardium had significantly increased LV filling pressures and decreased cardiac outputs. Similarly, we found significant differences in the LV filling pressure between the HFNEF patients and the normal subjects. 

We observed that the HFNEF patients had significantly lower global, apical, mid, and basal PSLSs than the normal subjects. Likewise, Saha et al.^41^ reported that the global longitudinal strain findings were significantly lower in the HFNEF group than in the normal group. Kraigher-Krainer et al.^34^ also noted lower longitudinal strain values for the HFNEF patients compared to the normal subjects in their investigation. In another study, Choi et al.^37^ observed that the mid and basal PSLSs in the patients with diastolic dysfunction grade I and II were significantly lower than those of the normal subjects. 

In the current study, a comparison of the correlation between the PSLSs and the markers of diastolic dysfunction demonstrated significant positive correlations between the global, mid, and basal PSLSs and the septal e' and negative correlations with the E/e' ratio. There were also negative correlations between the IVRT and the global and mid PSLSs and negative correlations between the NYHA functional class and the basal and mid PSLSs. It should be noted that most of the patients in our study had grade I diastolic dysfunction, which could be an important cause for the negative correlation observed between the STE findings. 

In contrast to our findings, Kraigher-Krainer et al.^34^ found no correlation between the longitudinal strain and the echocardiographic findings of diastolic function (e' or E/e'). In another study, Choi et al.^37^ observed that the mid and basal PSLSs had a significant correlation with the early diastolic mitral annular velocity, LAVI, e', and IVST, which had a close correlation with the diastolic function of the LV. 

The important finding in all these studies is that strain imaging using STE can determine some degree of systolic dysfunction despite a preserved LVEF detected by conventional echocardiography in HFNEF cases. 

The present study has some limitations. The study population is small and the percentage of the patients with higher grades of diastolic dysfunction is limited, with most cases having grade I diastolic dysfunction. Another weak point is that the patients’ drug history was not taken into consideration, which may have had some influence on the observed findings since many drug classes exert a significant impact on the systolic and diastolic functions of the LV, especially in ischemic and hypertensive patients. Enrolling more subjects with higher grades of diastolic dysfunction would be helpful. 

## Conclusion

The results of the present study showed that the HFNEF patients on conventional echocardiography had different STE findings compared to the normal subjects. The longitudinal systolic function of the LV, which was measured by STE, was reduced in all the segments, which was indicative of some degree of subclinical systolic dysfunction in these patients, although the global LVEF was preserved.

## Notes:

***This paper should be cited as:*** Toufan M, Gharebaghi SM, Pourafkari L, Delir Abdolahinia E. Systolic Longitudinal Function of the Left Ventricle Assessed by Speckle Tracking in Heart Failure Patients with Preserved Ejection Fraction. J Teh Univ Heart Ctr 2015;10(4):194-200.
